# Merkel Cell Polyomavirus and their Association with the Pathogenesis of Cervical Squamous Cell Carcinomas and Adenocarcinomas: A Review Article

**DOI:** 10.4314/ejhs.v33i4.18

**Published:** 2023-07

**Authors:** Hatim Makhdoom

**Affiliations:** 1 Applied Medical Sciences College, Laboratory Technology Department, Taibah University, Al-Madinah Al-Munwarah, Jeddah, north Obhur, Abdullah Althagfi

**Keywords:** Adenocarcinomas, Cervical Cancer, Merkel Cell Polyomavirus, Pathogenesis, Squamous Cell Carcinomas

## Abstract

**Background:**

This review aims to determine the potential role of Merkel Cell Polyomavirus (MCPyV) in the pathogenesis of cervical squamous cell carcinomas and adenocarcinomas.

**Methods:**

A PRISMA systematic search appraisal was conducted. The Scopus, Web of Science, PubMed, EMBASE, Google Scholar, and MEDLINE databases for publications in English were searched up to September 2022 for all relevant articles. All articles that have outlined the contributions of the MCPyV to cervical squamous cell carcinomas and adenocarcinomas were included.

**Results:**

The six databases produced 6806 articles. Only six articles met the inclusion criteria and were included. The protocol of this review was submitted and registered with the PROSPERO (Code no. CRD42022369197). The total sample size across the articles was 1135; the age of the participants ranged between 18 and 75 years. In addition, the included articles were conducted between 2012 to 2016. All included articles have a cross-sectional design.Furthermore, different kinds of samples were collected in the reviewed articles, namely cervical tissue biopsies, cervical smears, formalin-fixed paraffin-embedded resection specimens, and cervical adenocarcinomas. Moreover, five articles showed no statistically significant association between the MCPyV and cervical squamous cell carcinomas and adenocarcinomas. In contrast, one article revealed a positive association between MCPyV and cervical squamous cell carcinomas and adenocarcinomas.

**Conclusions:**

MCPyV could not be associated with the pathogenesis of cervical squamous cell carcinomas and adenocarcinomas. Further attention should be given to examining this association, and further studies with a large sample size are recommended to confirm these findings.

## Introduction

Cervical cancer is cancer arising from the cervix (1). It is due to the abnormal growth of cells that can invade or spread to other body parts (2,3). Worldwide, cervical cancer is the fourth most common cause of cancer and death from cancer in women (4). In 2018, over 300,000 cervical cancer deaths and an estimated 570,000 new cases (5). It accounts for about 8% of all cancer cases and all cancer deaths in women, making it the second most frequent cause of cancer in women after breast cancer (6). Cervical cancer is one of the leading causes of cancer death in low-income nations, affecting about 80% of cases (7). Furthermore, it is the most frequently detected cancer during pregnancy, with an occurrence of 1.5 to 12 for every 100,000 pregnancies (8).

Moreover, about 90% of cervical cancer cases are squamous cell carcinomas, 10% are adenocarcinomas (ACs), and a small number are other types (9). Usually, no symptoms are present at the beginning of cervical cancer. Atypical vaginal bleeding, pelvic pain, or discomfort during sex are possible later indications and symptoms (3). Even while post-sex bleeding may not be a serious issue, it could be a sign of cervical cancer. In advanced diseases, metastases may occur in the abdomen, lungs, or elsewhere (4).

The human papillomavirus infection causes more than 90% of cervical squamous cell carcinomas (SCCs) and ACs cases; other risk factors include smoking, a weakened immune system, birth control pills, beginning sex at a young age, and having numerous sexual partners, although they are less significant. In addition, human immunodeficiency virus (HIV) infection and genetic factors are contributed to cervical cancer risk (10,11,12,13).

Furthermore, papillomaviruses and polyomaviruses are small, spherical, nonenveloped, double-stranded DNA viruses that multiply in the nucleus. The two virus groups are unrelated (14-16). Papillomaviruses infect surface epithelia and produce disease at these sites. After multiplying at the point of entrance, polyomaviruses are transported by viremia to harm interior organs like the kidney and the brain. Viruses of both families produce experimental tumors in laboratory animals (17). In addition, the Merkel Cell Polyomavirus (MCPyV) belongs to the family Polyomaviridae. It is the only human polyomavirus (HPyVs) for which a strong correlation with human cancer, Merkel cell carcinoma, has been demonstrated. The MCPyV large T antigen (LT-Ag) can bind tumor suppressor protein pRb, a key regulator of cell cycle progression, and is a potential oncogene.

Moreover, truncation mutations in the viral LT-Ag gene result in replication incompetence and tumorigenesis (18). Additionally, to date, not all cervical cancer causes are known (19). Therefore, this review aims to determine the potential role of MCPyV in the pathogenesis of SCCs and ACs.

## Materials and Methods

**Article source and literature search**: The Preferred Reporting Items for Systematic Reviews and Meta-Analyses (PRISMA) criteria were followed in forming this systematic review (Table S1). The protocol of this review was submitted and registered with the International Prospective Register of Systematic Reviews (PROSPERO) (Code no. CRD42022369197). A general search was conducted in six major databases: Scopus, Web of Science, PubMed, EMBASE, Google Scholar, and Medical Literature Analysis and Retrieval System Online (MEDLINE) in order to locate information in English regarding the roles played by the MCPyV in the pathogenesis of SCCs and ACs without time restriction up to September 2022. Medical Subject Headings (MESH) and free-text search were the combined methods used in this review. A collection of search terms was produced using truncations, Medical Subject Headings (MESH), and Boolean operators (Table 1).

**Table 1:** Search terms and linkage (Medline)

Participants	exp patients with cervical cancer* OR cervical squamous cell carcinomas
	**AND**
**Exposure**	Merkel cell Polyomavirus* OR exp human Polyomavirus
	**AND**
**Outcomes**	exp Pathogenesis of cervical squamous cell carcinomas /OR
	adenocarcinomas OR exp Pathogenesis of cervical cancer

**Articles selection**: The prerequisites for inclusion were as follows: Articles have outlined the contributions the MCPyV makes to SCCs and ACs. However, the following exclusion standards were taken into account: (1) Reviews; (2) non-English language; (3) Case reports; (4) Articles not concentrating on the contributions of the MCPyV to SCCs and ACs; (5) Articles deficient in important information; and (4) Studies without full text.

**Data extraction**: Using the EndNote V.X8 program, the screening and article selection processes were controlled. After eliminating duplicates, the author individually reviewed the titles, abstracts, and complete texts to decide whether the papers were eligible. A standardized data collection form that was created following the sequence of variables required from the primary source was used to extract and record the first author's name, date of publication, article country, design, sample size, and characteristics, and the roles played by MCPyV in the pathogenesis of SCCs and ACs.

We could not do a meta-analysis since the outcome measures differed among the trials. Rather, narrative synthesis was carried out. Each article was introduced before being compared, analyzed, and then synthesized.

**Quality assessment of the included articles**: Since it is complete and enables a thorough review of the included research, an assessment tool was used. Utilizing “The Quality Assessment Tool For Quantitative Studies (QATFQS),” which was created by the “Effective Public Health Practice Project (EPHPP),” the included studies' quality was assessed (20). The eight components of the assessment tool that assess the study's quality include selection bias, study design, confounders, blinding, data collection procedures, withdrawals and dropouts, intervention integrity, and analysis. In order to determine the total research score, each component is given a score in one of three categories: “1 = STRONG (no ratings of WEAK), 2 = MODERATE (one rating of WEAK), and 3 = WEAK” (two or more WEAK ratings) (21).

## Results

**Figure 1:**
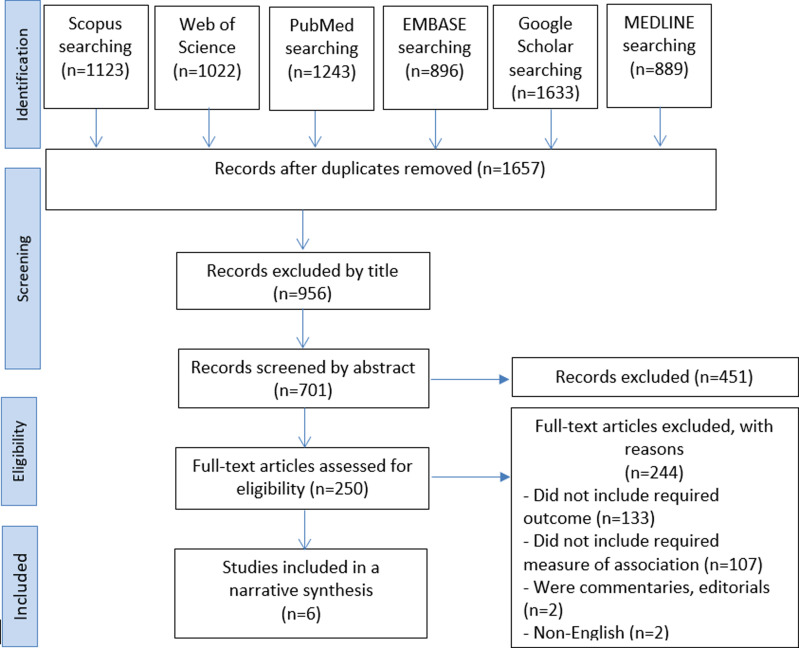
PRISMA flow diagram

The six databases produced 6806 articles as a result of the literature search. 956 of the 1657 unique articles were omitted from the remaining articles after 5149 duplicates were eliminated. After screening the remaining 701 articles based on the abstract, 451 were eliminated, leaving 250 articles. Two hundred forty-three articles were judged to be ineligible after reading the whole text, with the most frequent reasons being that they lacked the necessary outcome and measure of association. More information on the exclusion reasons is provided in the PRISMA flowchart (Figure 1). In the end, six articles in total qualified for the review.

**Descriptions of articles**: The six eligible and included articles are summarized in Table 2. The articles published in 2012 were conducted in Japan (22) and the United States (23). The article published in 2014 was conducted in Kenya (24) and China (25). The article published in 2015 was conducted in Iran (26), and the article published in 2016 was conducted in France (27). The total sample size across the articles was 1135. All of the six included articles were cross-sectional in design. Different kinds of samples were collected in the reviewed articles, namely cervical tissue biopsies (25, 26), cervical smears (23, 24, 27), and formalin-fixed paraffin-embedded resection specimens and cervical ACs (22). Different characteristics of recruited women across the included articles, namely women with cervical abnormalities that have been histologically proven (26), HIV positive and negative women (24), women with SCCs and women with cervical ACs (22), and women who underwent routine HR-HPV screening test (23). The ages of participating women ranged from18 and 75 years (24), 21 and 52 years (24), 28 and 74 years (22), and 26 and 70 years (23).

**Table 2:** Summary of the articles

Study	Country	Study design	Sample size	Age (years)	Sample characteristics	Type of specimen	Rate of positive Merkel cell Polyomavirus	Findings	Quality assessment
Salehi-Vaziri et al. 2015 (26)	Iran	A cross-sectional	112	Mean: 44.8±13.5	Women with cervical abnormalities	Cervical biopsies	33% (37/112)	NSSA with cervical cancer.	Strong
Alosaimi et al. 2014 (24)	Kenya	A cross-sectional	220	Median: 35	105 HIV-positive and 115 HIV-negative women	Cervical Smears	7.3% (24/329)	NSSA was found with cervical cancer.	Strong
Imajoh et al. 2012 (22)	Japan	A cross-sectional	64	Average : 43	Women with cervical SCCs and cervical ACs	Cervical SCCs and 16 cervical ACs samples	20.3% (13/64)	MCPyV was associated with cervical cancers.	Moderate
Fraase, K. et al. 2012 (23)	United States	A cross-sectional	249	Average : 45	Women underwent routine HR-HPV screening tests	Cervical swabs	6.4% (16/249)	NSSA with cervical cancer.	Moderate
Kolia-Diafouka et al. 2016 (27)	France	A cross-sectional	190	Median: 35.4	Women enrolled in the HARP (HPV in Africa Research Partnership) study	Cervical specimens	55.3% (105/190)	NSSA with cervical cancer.	Strong
Gu et al. 2014 (25)	China	A cross-sectional	294	NM	The JC and BK viruses infection was evaluated by detecting the viral DNA in samples	Urine, uterus, endometrial, and ovarian	NM	Only HPV infection is associated with cervical cancer.	Strong

*CIN2+: cervical intraepithelial neoplasia grade 2 or more severe; NM: None mentioned; NSSA: No statistically significant association*.

In addition, Table 2 shows the total number of cases across the articles was 1135; the type of specimens were cervical biopsies, cervical smears samples, tissue samples from cervical SCCs and 16 cervical ACs, cervical swabs, urine of myoma of uterus, cervical cancer, endometrial cancer, and ovarian tumor patients. Furthermore, the type of lesion (histology) were 30% squamous cell carcinomas, 37.5% ACs, and 16.7% undifferentiated carcinomas; cervical smears (6%) and cervical carcinomas (9%); 9/48 cervical cutaneous squamous cell carcinomas (SCCs) (19%) and 4/16 cervical ACs; BK Polyomavirus; and cervical intraepithelial neoplasia grade 2 (CIN2)+ (n= 54) and ≤ cervical intraepithelial neoplasia grade 1 (CIN1) (n= 51).

Moreover, the rate of positivity for Merkel cell analysis was 33% (37/112), 7.3% (24/329), 20.3% (13/64), 6.4% (16/249), and 55.3% (105/190), respectively.

Salehi-Vaziri, M. et al. 2015 reported that MCPyV infection had no statistically significant link to cervical cancer (OR, 1.12; 95% CI, 0.07-16.83) (26). Also, Alosaimi, B. et al. 2014 revealed the overall proportion of HPyVs positives in cervical smears (6%) and cervical carcinomas (9%), with no significant difference in HPyVs between these two groups (P-value > 0.05). As well, Fraase K. et al. 2012 concluded that there is no evidence of a link between co-infection with high-risk human papillomavirus (HR-HPV) and HPyVs (BK virus) in the cervical sample or a contribution from BKV to the development of cervical cancer (23). In addition, Kolia-Diafouka et al. 2016 showed no association between the detection of human polyomaviruses in cervical specimens and geographical origin/HIV serostatus, HR-HPV co- infection, or precancerous cervical lesion (27). Moreover, Gu et al. 2014 conclude that only HPV infection is associated with cervical cancer, and no synergistic effect on gynecological tumors caused by viruses' co-infection was observed. Also, JC virus infection is highly related to the pathogenesis of uterine fibroids (25). In contrast, Imajoh M. et al. 2012 showed that 9/48 SCCs (19%) and 4/16 cervical ACs (25%) were positive for MCPyV DNA and that MCPyV coexists in a subset of HPV-associated cervical cancers (22).

The quality assessment analysis showed that four articles were assessed as strong (23,24,25,26), whereas two articles were assessed as moderate (22,23) (Table 3).

**Table 3:** Quality assessment for the selected studies using The National Institute of Health quality assessment tool

	Salehi-Vaziri et al. 2015 (24)	Alosaimi et al. 2014 (22)	Imajoh et al. 2012 (20)	Fraase, K. et al. 2012 (21)	Ko-lia-Diafouka et al. 2016 (25)	Gu et al. 2014 (23)
**A**	2	2	3	3	2	2
**Selection Bias**						
**B**	2	2	2	2	2	2
**Study Design**						
**C**	2	2	2	2	2	2
**Confounders**						
**D**	2	2	2	2	2	2
**Blinding**						
**E**	1	1	1	1	1	1
**Data Collection**						
**Method**						
**F**	1	2	1	2	2	1
**Withdrawals**						
**And Dropouts**						
**G**	1	1	1	1	1	1
**Withdrawals**						
**And Dropouts**						
**H**	1	1	1	1	1	1
**Analyses**						
**Total**	1	1	2	2	1	1

*Note: Key answer: STRONG = 1; MODERATE = 2; WEAK = 3*

## Discussion

According to estimates, there were 604 000 new cases and 342 000 deaths from cervical cancer in women worldwide in 2020. In addition, about 90% of the new cases and deaths worldwide in 2020 occurred in low- and middle-income countries (28). The human papillomavirus infection causes more than 90% of SCCs and ACs cases; other risk factors include smoking, a weakened immune system, birth control pills, beginning sex at a young age, and having numerous sexual partners, although they are less significant. In addition, human immunodeficiency virus (HIV) infection and genetic factors contribute to cervical cancer risk (10,11). Two HPV types (16 and 18) are responsible for nearly 50% of high-grade cervical pre-cancers (10,11).

Furthermore, 5% of all cervical cancer cases are thought to be related to HIV, and women with HIV are six times more likely to acquire cervical cancer than women without HIV (27,29).

Additionally, in every part of the world, younger women are disproportionately affected by HIV's link to cervical cancer (28,30). Programs are in place in high-income nations to allow girls to receive the HPV vaccine and women to receive frequent screenings and appropriate care. Precancerous lesions can be found through screening at an early stage when they are still treatable (29). These prevention methods are limited in low- and middle-income countries, and cervical cancer is frequently not discovered until it has progressed and symptoms appear. Additionally, these nations may have restricted access to treatments for cancerous lesions, such as cancer surgery, radiotherapy, and chemotherapy, contributing to a greater rate of cervical cancer death (7).

Recent reports show that papilloma and polyomaviruses produce experimental tumors in laboratory animals (17). In addition, six of the HPyVs have been associated with human diseases, including cancer (18). Moreover, not all cervical cancer causes are known (19). Therefore, the current review aimed to determine the potential role of MCPyV in the pathogenesis of SCCs and ACs. To the best of our knowledge, this is the first review that shows this association.

In the current review, the PRISMA systematic search appraisal was conducted for publications in English up to September 2022 for all relevant articles. All articles that outlined the contributions of the MCPyV to SCCs and ACs were included. In addition, the six databases produced 6806 articles as a result of the literature search. Only six articles met the inclusion criteria and were included in the final analysis. The total sample size across the articles was 1135. The age of the study participants ranged between 18 and 75 years. Further studies are recommended to explore the role of young ages and double viral positivity and their association with cervical oncogenesis. Furthermore, all included articles have a cross-sectional design.

Additionally, different kinds of samples were collected in the reviewed articles, namely cervical tissue biopsies, cervical smears, formalin-fixed paraffin-embedded resection specimens, and cervical ACs. The main results of this review demonstrated that five out of six included articles showed no statistically significant association between the MCPyV and SCCs and ACs. In contrast, one article revealed a positive association between the MCPyV, SCCs, and ACs.

The first human polyoma viral pathogen identified was MCPyV. Most instances of Merkel cell carcinoma, a rare but aggressive kind of skin cancer, are thought to be caused by it. Additionally, MCPyV infection has been discovered in almost 80% of Merkel cell carcinoma tumors. Since it is present in respiratory secretions, the respiratory system may be the transmission route. But because HPV can also be present in healthy skin, gastrointestinal system tissues, and other places, it is unclear exactly how it spreads (31). A recent study suggests that MCPyV-positive tumors have a better prognosis than uninfected tumors. Routine virus detection may benefit medical guidance in the future (32). In addition, the HPyVs 2, commonly called the JC virus, is a type of human polyomavirus. Several reports have postulated that JCV infection is associated with various types of human cancers, including gastrointestinal, colorectal, gastric, esophageal, and brain (33,34). The debate about polyomaviruses in human cancer arose from the finding that polyomaviruses can transform cells and are oncogenic in non-permissive hosts (35).

Furthermore, the polyomavirus hominin 1, also called BKV, is well-known as a principal predisposing factor for different cancers (36). Few studies show the role of BKV in cervical cancer, which makes the comparison more difficult. On the contrary, in the current review Imajoh, M. et al. 2012 showed that 9/48 SCCs (19%) and 4/16 cervical ACs (25%) were positive for MCPyV DNA and that MCPyV coexists in a subset of HPV-associated cervical cancers (20). The role of MCPyV in the pathogenesis of SCCs and ACs needs more study.

The main strength of the current review is the first review that shows the role of MCPyV in the pathogenesis of SCCs and ACs. However, this review has several limitations. All included articles have a cross-sectional design; the causal relationship could not be determined, limiting the generalizability of the review findings and the small sample size. Furthermore, the author individually reviewed articles to decide whether the papers were eligible, which was another limitation. Additionally, we could not do a meta-analysis since the outcome measures differed among the studies.

In conclusion, the MCPyV could not be associated with the pathogenesis of SCCs and ACs. Further future studies with a large sample size are recommended to confirm these findings. Further attention should be given to investigating the MCPyV and their association with the pathogenesis of SCCs and ACs. In addition, comprehensive cervical cancer control programs, including primary prevention, secondary prevention, tertiary prevention, and palliative care, should be implemented to reduce the high rate of SCCs and ACs, especially among young women.
